# The politics of bailouts: Estimating the causal effects of political connections on corporate bailouts during the 2008–2009 US financial crisis

**DOI:** 10.1007/s11127-020-00871-w

**Published:** 2021-02-06

**Authors:** Vuk Vukovic

**Affiliations:** 1 Department of Politics and International Relations, University of Oxford, Manor Road Building, Manor Road, Oxford, OX1 3UQ UK; 2 Oraclum Intelligence Systems, 23 Arnold Close, Hauxton, Cambridge, CB22 5FN UK

**Keywords:** Bailouts, Political connections, Lobbying, Campaign spending, US financial crisis, TARP, D72, H81, G01

## Abstract

In 2008, as the financial crisis unfolded in the United States, the banking industry elevated its lobbying and campaign spending activities. By the end of 2008, and during 2009, the biggest political spenders, on average, received the largest bailout packages. Is that relationship causal? In this paper, I examine the effect of political connections on the allocation of funds from the Troubled Asset Relief Program (TARP) to the US financial services industry during the 2008–2009 financial crisis. I find that TARP recipients that lobbied the government, donated to political campaigns, or whose top executives had direct connections to politics received better bailout deals. I estimate regression discontinuity design and instrumental variable models to uncover how election outcomes for politicians in close races affected the distribution of bailout funds for connected firms. The results do not imply that some banks were deliberately favored over others, just that favored banks benefited because of their proximity to the right people in power. If being politically connected matters in general, in times of crisis it matters even more.

**Supplementary Information:**

The online version contains supplementary material available at 10.1007/s11127-020-00871-w.

## Introduction

The biggest financial crisis since the Great Depression started with the collapse of the US housing market in 2007, which subsequently exposed systemic risks characterizing the US finance industry. The bankruptcy of Lehman Brothers in September 2008 triggered a downward spiral of financial institutions in desperate need of refinancing to avoid the same scenario. Policymakers responded, and in October 2008 drafted the Troubled Asset Relief Program (TARP) that would over the next year provide liquidity to distressed financial institutions. Total government spending under TARP was $372 billion (2.55% of US GDP). At the same time, in 2008 and 2009, lobbying expenditures and campaign contributions of TARP recipients increased significantly. Conventional wisdom would suggest a connection between TARP funds and lobbying and donations. The literature that links lobbying to the distribution of TARP funds (e.g.,  Duchin and Sosyura 2012; Igan, Mishra and Tressel 2012; Blau et al. 2013; Calomiris and Khan 2015; Blau 2017), mostly using some type of multivariate analysis, finds that more lobbying effort increased the probability of bailouts. Those conclusions, however, could be misleading because they are not robust to endogeneity issues like self-selection or omitted variable bias. None of the studies claim to have confirmed a causal relationship, meaning that any conclusion about a relationship between bailouts and political influence requires further scrutiny.

Fortunately, that episode of US history provides an opportunity for a compelling research design that is able to address endogeneity issues and estimate the causal effect of political connections on the distribution of TARP funds, at least among its politically connected recipients. As the crisis was unfolding and politicians needed to make a succession of quick decisions on how to deal with the ostensibly illiquid banking system, the United States was in the midst of a national political campaign for the next president and seats in the new 111th Congress. That context, having a federal election right in the middle of the biggest banking panic since the 1930s, allows me to exploit close electoral races as sources of as-good-as-random assignment of politicians connected to TARP recipients, and helps answer the main question of interest, namely, did political connections matter in the distribution of TARP funds?

**Fig. 1:**
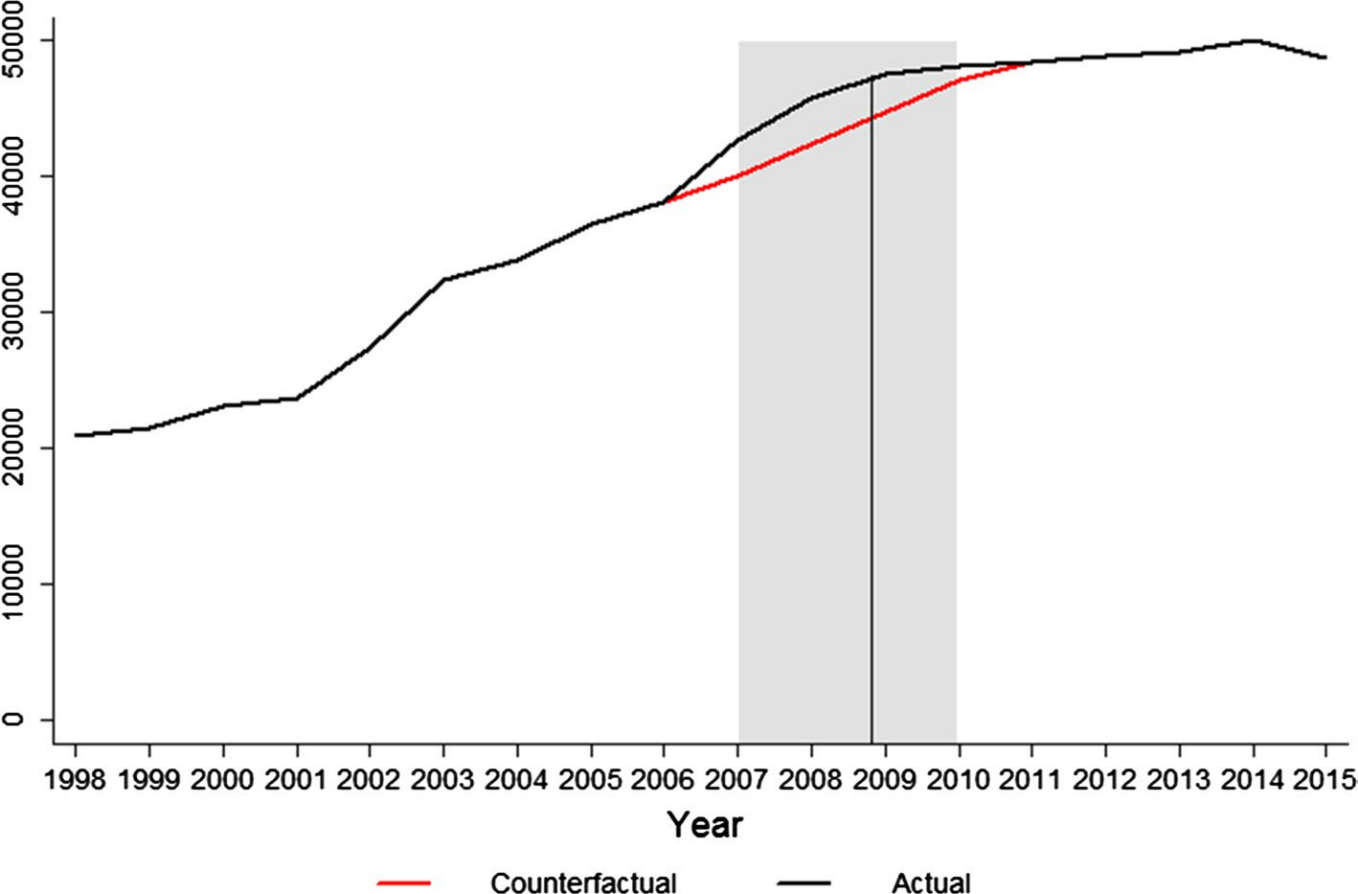
Actual (black) and counterfactual (gray) inflation-adjusted lobbying spending of financial firms (*y*-values in millions of dollars). Counterfactual lobbying is the estimated size of lobbying expenditures had it not been for the crisis. It is estimated using simple exponential smoothing, according to Eq. 1 described in Sect. 4. The difference between actual and counterfactual lobbying in 2008 and 2009 is what is defined to be *abnormal lobbying*. The straight vertical line denotes the start of the bailout allocation process (October 28, 2008)

**Fig. 2:**
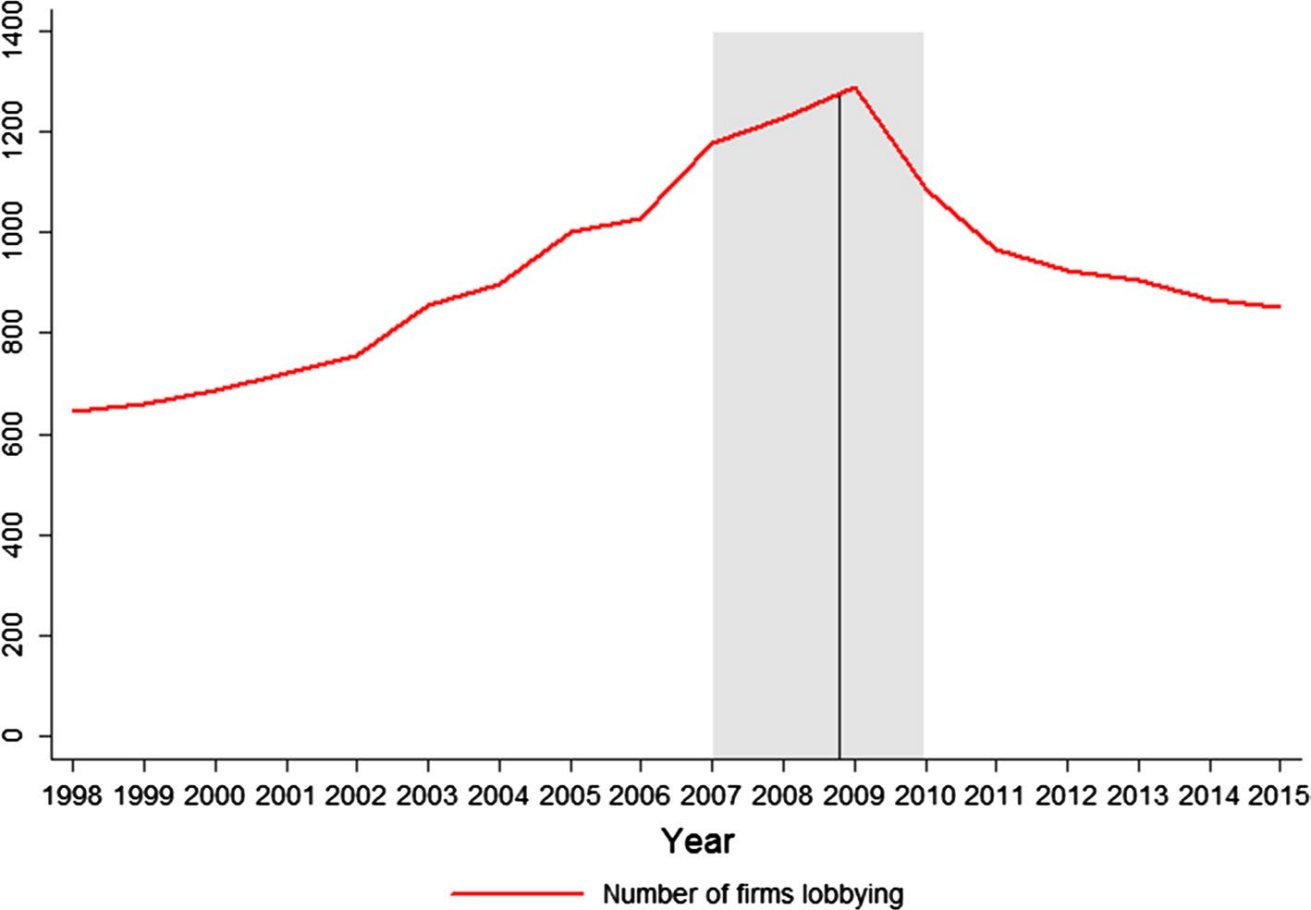
Total number of financial services firms lobbying, peaking in 2009. The straight vertical line denotes the start of the bailout allocation process (October 28, 2008)

Consider Figs. 1 and 2, showing a peak in industry lobbying and the number of financial firms that lobbied during the distribution of TARP funds in 2008 and 2009. The figures reveal that the upward trend of lobbying started several years before the crisis, raising doubt about the potential causal mechanism of 2008 and 2009 lobbying on bailout allocations suggested by conventional wisdom. Furthermore, large within-industry variation is obvious. Some firms lobbied and contributed to political campaigns more than others, some had stronger political connections, but some also were much more exposed to risk to begin with. Saying that firms lobbying more got more money from bailouts does not determine whether political connections actually *caused* better bailout deals. A large number of potential confounders could have affected the distribution of TARP funds, but had nothing to do with political connections, lobbying, or campaign financing. Therefore, any research that seeks to uncover the true causal implication underlying the possible relationship must solve two basic methodological problems. First, a self-selection problem—firms are more likely to lobby and donate to campaigns if they expect successful outcomes and a return of favor from the politician. Second, omitted variable bias may arise. The driver of the variation that increased lobbying spending and the likelihood of getting a bailout simultaneously could be banks’ risk exposures. Riskier firms lobby and donate more because they are more prone to risk, implying they also will have a greater chance of getting bailout funds. Exposure to risk, not political spending, thus could be the main explanation for bailout allocations.

The central motivation of the present study is to eliminate all such potential alternative explanations and at the same time mitigate concerns about all other endogeneity issues affecting the relationship. Accordingly, the paper delivers two important contributions to the literature. First, it captures three potential sources of political influence. It examines the impact of financial firms that either lobbied the government in 2008 and 2009, made campaign donations in the 2008 election cycle, or whose upper-management executives held high-level positions in government at least five years prior to the crisis. All three types of political influence need to be accounted for in order to measure the full effect of political connections on the allocation decisions of the US Treasury and Congress, and to see whether those connections swayed more funds towards better-connected firms.

Second, in order to verify the causal effect of political connections on bailout allocations and resolve the aforementioned endogeneity issues, I apply two methodological approaches based on natural experiment designs: a regression discontinuity (RD) approach and an instrumental variables (IV) approach. I look at the allocation of bailout funds to firms whose connected politicians lose a very close election (decided within a 1% margin of victory). Both empirical approaches draw randomization from the assumption that in such close races a random element such as luck is most likely to have tipped the outcome, meaning that bare winners and losers—and firms attached to them—are supposed to be statistically interchangeable.

The sample consists only of firms that received TARP funds, separated into connected and unconnected firms, meaning that all of the estimated effects necessarily are local treatment effects. The general research question is whether political connections result in larger bailout packages for firms that received TARP funds. The RD and IV analyses are more specific. They restrict the sample to connected firms whose politicians won narrow elections. The RD estimation finds that a connected politician’s close electoral victory increased the bailout allocation for that politician’s connected firm by around 13%, on average. The IV approach adopts an instrument that is a variation of the RD version: instead of connected firms per politician, I look at the number of connected politicians winning close elections for each firm. The estimated effect is then the impact of one additional connected politician who won a close election on the size of bailout received. The IV approach finds that firms with more connected politicians who won closely contested elections received more TARP funds (the effect is a 19% increase in TARP funds for a one-standard-deviation increase, which is about 12 more connected politicians winning close elections). These are two different effects, but they both suggest a similar conclusion: being politically connected clearly made a difference in the allocation of TARP funds among those financial institutions who received them.

The paper therefore is the first to examine political connections in the context of the 2008–2009 bailouts using close elections in order to draw stronger causal inference claims about their relationship. It differs from the existing literature in two other aspects. Studies looking into the relationship at hand typically examine all firms that were eligible to receive TARP funds, whereas the present paper focuses only on a narrow sample of statistically interchangeable recipients, comparing firms based on the electoral successes of their connected politicians. Finally, prior studies typically focus only on lobbying activities and, to a lesser extent, campaign spending as measures of political influence. This paper expands the definition of political influence to include three different forms of political connections: lobbying effort, campaign contributions, and direct personal connections between financial institution executives and legislators. That expanded definition enables me to capture more accurately different elements and patterns of political influence and to examine a wider implication of political connections on government funding allocations.

The study thus aims to provide a blueprint for further research on political influence and the distribution of government funding, particularly during times of high-stress events or serious economic hardships. For example, at the onset of the COVID-19 pandemic, many governments offered financial assistance to firms as ways of helping them survive the shock. A researcher looking to examine a potential impact of political connections on the allocation of such funds needs to find a good source of exogenous variation to test such a hypothesis. Any country that held elections during the allocation process would be a good place to start.

The next section describes the context of the US financial crisis and the TARP allocation process. To understand the process fully, I relied on information from interviews with former employees of the Office of the Special Inspector General for the TARP, the US House Oversight Subcommittee on TARP, and the US Treasury. Section 3 describes the sources of data and defines all variables. Section 4 outlines the empirical strategies adopted to examine the relationship of interest and presents the results. The final section concludes by discussing the validity of the results obtained by two different methods.

## Political connections and bailouts in the context of the 2008–2009 US financial crisis

### The allocation of TARP funds

The selection criteria for TARP was based on the CAMELS rating system, which ranked all US financial institutions based on how likely they would be to need recapitalization using a weighted index of their various risk and performance indicators.^1^ The decisions about eligibility and selection for funding were made by the Treasury using CAMELS ratings, in consultation with the Federal Reserves. Eligible institutions could apply for assistance and the Treasury could accept or reject the application. Receiving TARP funds had its downsides as well—it was subject to limits on executive pay, bonuses, and golden parachutes, in addition to losses of some tax benefits. However, perceptions arose that the selection criteria were not clear enough and that too much power was given to the Treasury (Veronesi and Zingales 2010), that it favored institutions that would survive anyway or were too “big to fail” (Dash 2008), and that some members of Congress were funneling resources to banks in their own districts (Pana and Wilson 2012).

According to Duchin and Sosyura (2012), out of 537 publicly traded firms that were eligible for the Capital Purchase Program (CPP)^2^ (the total number exceeded 900 institutions, but the authors focused only on the subsample of public firms), 80% applied for the program, most of which were approved and received funding (only 15% rejected it after being approved). Participating in TARP therefore was attractive for most firms, despite limits on executive compensation. Some banks even falsified financial statements in order to be eligible for the program, falsifications that later were uncovered by the Special Inspector General for the TARP in his report to Congress (Special Inspector General for the Troubled Asset Relief Program 2011). Even though some financial institutions wanted to get out of TARP already in mid-2009—to remove the stigma associated with participating in the program—the motivation to be included in TARP was to mitigate uncertainty at the height of the crisis. During uncertain times, when industry-wide panic sent shock waves throughout the economy, requesting TARP funding was the best available strategy a bank could take advantage of to remain solvent.

Mian et al. (2010) have shown that campaign contributions from the financial services industry in 2008 strongly predict the votes of individual members of Congress in favor of the Emergency Economic Stabilization Act (EESA).^3^ Therefore, despite their later pushback, the majority of banks eagerly participated, which induced them to activate their connections to politics, either through abnormal increases in lobbying, campaign spending, or direct connections to their former colleagues at the Treasury, Congress, or the Fed. It is for those reasons that I focus only on firms that received bailout funds, instead of comparing recipients and non-recipients. The majority of non-recipients did not have incentives to participate, so it would be misleading to compare their outcomes to the recipients, not to mention that doing so could bias the estimates.

Finally, prior research clearly suggests that individual members of Congress exerted some sort of influence over the entire process (Mian et al. 2010; Pana and Wilson 2012), implying that even though the final decision about what institutions receive funding was made by the Treasury following a clear-cut formula, the decision itself was subject to influence by a number of actors. Financial institutions had two ways of trying to influence funding allocations: they lobbied or donated campaign funds to members of Congress (including both House and Senate members), or they exploited their personal connections to anyone inside the Treasury, the Fed, or Congress to try to get better deals for themselves. Influencing members of Congress was most likely motivated by encouraging them to vote for the TARP. However, given that some congressmen were shown to be favoring their districts, they too influenced the decision-making process, despite not being involved directly in funding allocations. How personal connections were used to make it happen is described in the following section. The empirical logic of the paper relies on the decision-making mechanism, such that the process itself potentially was subject to outside influence.

### The role of personal connections

The context of the crisis was important because it reveals a very specific way in which policymakers interacted with industry giants in order to moderate the crisis. Lobbying and political spending on the part of the financial services industry at the time were examples of both intra-industry and inter-industry competition for political favors. At the height of the crisis in September and October 2008, while TARP was being conceived and drafted, the chief executives of the eight biggest financial institutions in the country held regular formal and informal meetings with New York Fed Chairman Timothy Geithner and Treasury Secretary Henry Paulson (Becker and Morgenson 2009; Stewart 2009), both of whom had strong ties to the banking industry throughout their earlier careers. The very connections the banks had with those individuals suggest a cartel model of industry-wide influence over the policymaking process. That high-profile example is not modeled explicitly in the paper given that its focus is on the reverse relationship—corporate executives who once occupied governmental office—however, the case exemplifies an elite network forged between industry giants and its key regulators.

Taking advantage of connections, lobbying, or campaign spending to get favorable treatment from the government is not inherently illegal. Acemoglu et al. (2016) examined the impact of connections of firms to Treasury Secretary Timothy Geithner during the crisis, finding that firms connected to Geithner earned abnormal market returns immediately after his nomination by the president-elect. They called that effect the “connections in a crisis hypothesis”—during crisis times it is natural for decision-makers to seek advice from within their own social networks. Similarly, Querubin and Snyder (2013) report that rent-seeking tends to become more active during episodes of great political or economic turmoil. The explanation is that government expenditures increase rapidly during crises, and media oversight is less effective. If members of Congress or the Treasury Secretary himself tapped their own networks in an attempt to get better information or simply decide how to approach a given issue, then it should not be surprising to see that well-connected firms received better bailout deals. The mere persistence of social interactions with decision-makers is enough to establish the effect. Similar to the concept of cultural capture in financial regulation (Kwak 2014),^4^ the decision-makers at the Treasury and in Congress were influenced by their own connections.

The joint efforts of the Fed, the Treasury, and big bank CEOs to find a solution to the liquidity and solvency crisis created a lifeline for many other financial institutions down the line who then utilized their own political connections to get better parts of the bargain. The policymakers’ reaction to the crisis, arguably influenced by their within-industry personal networks, created a positive externality for 900 banks and other financial institutions that received bailout funds. That is where the inter-industry competition for bailout allocations started. The stage was set for massive campaign spending and lobbying by an unprecedented number of financial institutions (see Fig. 2) to ensure that each received its own piece of the pie. The personal connections of the biggest banks’ CEOs with senior regulators set the deal in motion, after which lower-level personal networks were activated by individual banks to gain a short-run government stimulus. Individual firms that cultivated relationships with politicians should have secured better deals than firms having no such connections.

## Data and variables

### Bailout data and covariates

The dataset was assembled from three separate sources. I first gathered data on total bailouts received from the US Department of the Treasury’s (2013) Office of Financial Stability and its information on the total distribution of TARP funds from November 2008 until December 2009. These data include the Capital Purchase Program (CPP) worth $205 billion in total, as well as all other spending allocated to banks (commercial and investment), mortgage lenders, credit unions, insurance companies, financial services companies, and the rest of the financial industry. The total bill was $623 billion; however, I exclude government-sponsored enterprises (Fanny Mae and Freddy Mac) and automobile companies (GM and Chrysler). That brings the sample’s outlays down to $372 billion, allocated during the stipulated period to 962 financial institutions.

This initial dataset then was merged with information on bank balance sheets from the Federal Deposit Insurance Corporation (2016) quarterly Uniform Bank Performance Reports (“call reports”). The merger was performed by institutional name and by state; however, I cross-referenced most of the entries manually owing to different bank names in the two datasets and because the Treasury dataset did not provide bank addresses or postcodes. The finally assembled dataset enabled me to extract for each bank its total assets, total liabilities, total deposits, net loans and leases, Tier 1 capital, net risk-weighted assets, total equity, cash holdings, net income, earnings, assets, allowance for loan and lease losses (ALLL), total debt, employee salaries and benefits, and total number of employees. From that information, I managed to construct the main dependent variables: the natural logarithm of total bailout funds—*Log bailouts* ($\log B_{i}$)—and the ratio of *bailout funds to total assets* ($\frac{B_{i}}{A_{i}}$) for each firm *i*, in addition to a number of financial indicators useful in evaluating bank business performance and, what is more important, their exposure to risk. The list of all covariates (performance indicators, risk indicators such as the CAMELS rating) and how they were calculated is available in the Online Appendix.

The FDIC’s call reports only cover banks, and contain no data on the balance sheets of mortgage companies, insurance companies, investment funds, or other financial service companies, all of which received considerable sums as part of TARP. In order to gather data on other companies that received bailout funds, I relied on the official reports of the Securities and Exchange Commission (2017) for each institution that was a publicly listed company. That was done manually for 52 institutions. The final sample, after merging and manually cross-referencing, included a total of 685 financial institutions that received bailout funds in the total sum of $329 billion, or 88% of the original financial institution sample (excluding GSEs and automobile companies).

I created two main datasets for two different estimation strategies. The first was created by merging financial institutions’ balance sheet data from the FDIC and the SEC with the bailout data from the Treasury, as well as the lobbying, campaign contribution, and political connection variables for each firm. That sample allowed me to construct a new treatment variable and a corresponding instrument for two-stage least squares (2SLS) estimation. The second dataset comprises a subset of firms that had political connections (264 of them) and connects them to all politicians to whom they donated money during the 2008 election campaign, lobbied directly, or had personal connections with in order to estimate the regression discontinuity model. Given that many firms were connected to more than a single politician, that procedure resulted in a sample size of 2650 observations. I cross-referenced all politicians who had connections to an individual firm manually to avoid any mistakes. Next, I allocated each politician to his or her 2008 election results (whether they won or lost, and by what margin), their districts, the rate of mortgage foreclosures in each district, and other personal characteristics (e.g., party, age, and gender). Those data were obtained from the Federal Election Commission (2009) for the 2008 House and Senate elections, while the foreclosure data were taken from the US Department of Housing and Urban Development (2010). Summary statistics for all variables are reported in the Online Appendix.

### Measuring lobbying and political connections

The explanatory variables were taken from two separate sources. The lobbying and campaign spending data were downloaded from the online database at the Center for Responsive Politics (CRP) (2017).^5^ That source contains total lobbying expenditures and campaign spending for each firm in the financial services industry for a given year, as well as a directory of all politically connected lobbyists.

For the political influence variable, I accessed the BoardEx database, produced by Wharton Research Data Services (2017).^6^ The two datasets were relied on to create a unique political connections (*PolCon*) variable attempting to capture several levels of potential political influence: lobbying just before and during the bailout, campaign spending in the 2008 cycle, and the personal connections of top firm executives to decision-makers. I summarize the intuition behind the inclusion of each sub-variable.

The BoardEx database allowed me to access career histories and trajectories of more than one million senior corporate executives and determine which of them had direct connections to either the Treasury, Congress, or the regulators (the FED and the SEC in this case). A bank executive—defined to include any upper management director position, CEOs, CFOs, COOs, chairmen, presidents, and members of boards of directors—is coded as connected if he or she held a position at the Treasury or a relevant industry regulator (the Fed or the SEC), or had served in Congress, either as a formerly elected politician or a senior staffer, at least five years prior to the crisis. The justification for using such a narrow time span is that anyone holding a senior (top-level management) position at a financial institution is highly unlikely to have been a low-level civil servant recently before his or her current job. Senior bank executives who had experience in government were in each case employed previously in decision-making public sector positions before arriving at their current executive jobs. During their times in public office, they were very likely to have had personal relationships with people who were decision-makers during the bailout process. That sub-variable therefore captures whether or not a firm employed anyone in upper management who could have fostered close relationships with decision-makers in Congress or the Treasury.

In addition to direct political connections, I also look at bank lobbying and campaign expenditures in the 2008 election cycle. Using the CRP’s (2017) databases on lobbying and political contributions, I design the following two sub-variables: 2008 cycle *Campaign spending* and *Abnormal lobbying*. The *Campaign spending* variable is self-explanatory: it looks at firms that made political contributions in the 2008 election cycle and how much money they spent.

The *Abnormal lobbying* variable requires further explanation. Unlike some previous studies that focused on the total amount of lobbying in the years before the crisis—e.g., Igan et al. (2011) rely on lobbying expenditures from 2000 to 2006, while Blau et al. (2013) use lobbying expenditures from 2004 to 2008—I look at the immediate responses of lenders to the crisis shock and focus only on excess or abnormal lobbying done in 2008 and 2009. In the decade before the crisis, the financial industry’s lobbying efforts were aimed predominately at attaining more lenient regulations, which could have increased the entire industry’s appetite for risk-taking. Including pre-crisis lobbying that increased industry risk-taking could bias the estimated effect of lobbying on bailouts upwards given that more risk exposure motivated the size of the bailout package. In other words, a positive effect of lobbying on bailouts would not necessarily be driven by more lobbying, but by greater risk-taking. In order to eliminate that concern, I exclude all pre-crisis lobbying and focus only on *excess* lobbying in 2008 and 2009, narrowing the interest down to a single mechanism: the immediate strategic responses of firms to the financial panic. Did firms react by lobbying more and for different favors than they would have otherwise, and did that help them secure better bailout deals?

The variable is defined based on Jayachandran’s (2006) calculation of abnormal market returns, where *Abnormal lobbying* represents the difference between the *expected mean* of lobbying expenditures (the counterfactual level of lobbying—shown as the red line in Fig. 1) and the *mean of actual lobbying* expenditures in 2008 and 2009. The expected (counterfactual) levels of lobbying for 2008 and 2009 were predicted using simple exponential smoothing.^7^ The formula used to calculate *Abnormal lobbying* then is:

1 $$\begin{aligned} AbnL_{it}=L_{it}-E(L_{it}) \end{aligned}.$$

The main explanatory variable *PolCon* is coded as an indicator variable, where $PolCon=1$ if a firm (a) engaged in abnormal lobbying during 2008 and 2009, (b) funded any political campaigns during the 2008 election cycle, or (c) had any senior executives who used to work at the Treasury, in Congress, or for a financial industry regulator in the five years prior to the crisis. In total, 264 such firms are in the sample. A potential conceptual problem arises here because three different variables (lobbying and campaign spending are not equivalent) are pooled into one binary indicator. However, I ran all regressions using each individual variable, both as an indicator and as relative or absolute values of lobbying and spending; they all suggest the same conclusion, so the decision to pool them together is out of mere simplicity.^8^

Table 1 reports descriptive statistics for the distribution of *Share of bailouts in total assets* and *Log bailouts*,^9^ both with respect to the indicator variable of political connections *PolCon*. It also shows a *t*-test for the comparison in means between connected and unconnected firms for both dependent variables. A clear difference emerges in the distribution of bailouts for firms that were connected and firms that were not. A simple comparison of means shows that politically connected firms had a 2.4-percentage-point higher share of bailouts in total assets than non-connected firms, which constitutes a large effect as can be inferred from the first row of Table 1. However, that comparison still tells us nothing about any other unobserved (firm-specific or politician-specific) characteristics that could have driven the allocation of bailouts. In order to make the comparisons more realistic and in order to draw some inferences from them, I adopt two main empirical strategies, described in the following section.

**Table 1:** Descriptive statistics of the main dependent variables with respect to political connections

	Mean	Min	25th pctl	Median	75th pctl	Max	*N*
*Entire sample*							
Bailouts to assets	0.04	0	0.02	0.024	0.028	0.96	685
Log bailouts	16.4	7.4	8.47	16.2	17.3	24.9	679
Politically connected = 1							
Bailouts to assets	0.055	0.0006	0.02	0.025	0.03	0.96	264
Log bailouts	17.9	11.3	16.67	17.54	18.8	24.9	264
*Politically connected = 0*							
Bailouts to assets	0.031	0	0.19	0.024	0.027	0.9	421
Log bailouts	15.46	7.4	14.9	15.6	16.3	18.7	415

*t*-test connected vs. unconnected				*t*-test connected vs. unconnected			
(Bailouts to assets)				(Log bailouts)			
0.0239***				2.435***			
(0.0063)				(0.138)			

## Empirical strategy and results

The goal of the paper is to uncover the extent to which political connections mattered in the allocation of bailouts to TARP recipients. The focus is on TARP recipients only, i.e., banks and financial institutions that applied for taxpayer-financed relief. The counterfactual scenario is how much a TARP recipient would have received had it not been politically connected, while the question of interest is whether being politically connected helped a TARP recipient secure a better deal for itself. However, the analysis is not as straightforward as it seems, primarily because the allocation of TARP funds was not random, nor did firms assign themselves randomly into connected and unconnected categories. In order to establish the existence of any potential causal relationship between political connections and bailouts, and thus address the endogeneity concerns, I adopt two methodological approaches.

### Imposing randomization: regression discontinuity design

One way to solve the endogeneity problem is to exploit some arbitrary or rule-based threshold that allows as-if-random assignment of units into treatment and control groups around the threshold. Fortunately, the majority of bailout funds were distributed after the 2008 national elections (see Timeline in the Online Appendix), meaning that the discontinuity that could be exploited in the case at hand is the 50% vote share that determines the winners or losers of electoral races. Much of the literature relies on the fact that very narrow races around the 50% voting threshold (someone winning by 51% to 49%) are reasonable sources of as-good-as-random variation given that in such close races, luck is likely to affect the outcome (Lee 2008; Dal Bo et al. 2009; Eggers and Hainmueller 2009; Brollo and Nannicini 2012; Boas et al. 2014).

As-good-as-random variation of electoral outcomes within a narrow interval of the 50% cutoff implies that winners and losers of such races should be similar in all unobservable characteristics (e.g., ability, appearance, successful campaigns) because the electoral outcome itself was more a result of luck than ability, skills, or any other factor that we *can* measure like campaign funding or incumbency status. The closeness of the race is the source of random variation which firms cannot control, meaning that the electoral outcomes of politicians tied to firms are therefore exogenous to the firms. In that way I eliminate the influence of all unobservable factors that might affect TARP allocation decisions, be they firm-specific or politician-specific. The focus is given to narrow, as-if-random exogenous events (close elections), which set the dataset up as a quasi-experiment wherein I compare bailouts given to firms connected to winning politicians versus bailouts given to firms connected to losing politicians.

The unit of analysis necessary for that strategy is firm-politicians. I assign all *connected* US Representatives and Senators to each firm as new individual observations, thus expanding the overall sample size to 2650 observations (see the data section and the Online Appendix for summary statistics) and, consequently, the variation in the number of politicians winning or losing close races. The connected members of Congress in the sample are those who were connected to firms through any of the *PolCon* sub-variables: if they had direct connections to executives, if they were lobbied (most applicable to the members assigned to specific committees), or if they received campaign donations from financial sector firms in the 2008 election cycle. Defining the sample in that way allows me to exploit as-if-random assignment of winners and losers in close congressional races to see how a win or a loss of a *connected* federal legislator affected the sizes of bailouts to the connected firm. If a member of Congress barely won his or her seat, all firms that lobbied or donated money to that politician should receive larger bailouts than firms that lobbied or donated to a politician who barely lost.

Obviously, firms donate and are connected to multiple candidates in a given election cycle, meaning that the source of variation comes from differences across firms. The sample for regression discontinuity design (RDD) is defined by assigning individual politicians to firms, meaning that clusters of units are assigned to the treatment group, necessitating the clustering of standard errors within firms in order to reduce the errors across observations. The estimated coefficient should therefore be interpreted as the effect of an additional recipient candidate on TARP bailouts for a firm, holding all other candidates’ electoral performance constant. The outcome variables are: *Share of bailouts to total assets* and *Log bailouts* given to all firms connected to an individual politician.

Given that elections were held on November 4th, 2008, and that the vast majority of bailout allocations were awarded after the election (see Timeline in the Online Appendix), the probability of post-treatment bias is reduced. The EESA was voted into law by the previous Congress, before the election, but the allocation decisions on who got funds weren’t made until after the election for more than 95% of all benefitting financial institutions. The exceptions were AIG, which received help on several occasions, and the eight largest banks,^10^ which already had agreed to initial bailout packages on October 14th, immediately after the announcement of the CPP (and received the funds on October 28th). In order to directly account for that, I exclude campaign donations and lobbying spending of the eight big banks, except for AIG, Bank of America, and Citigroup, all of which received additional funds after the election. The outcome variables for those institutions are adjusted to account for only the newly received bailout funds. Doing so creates my *reduced* sample, whose results are presented alongside the *full* sample ones. For a deeper discussion of why those big banks should be included, see more in the “Notes on data” section in the Online Appendix.

As specified, the RD approach depends on exploiting an arbitrary rule to create a quasi-experimental setting. In the case of two-party elections, the cutoff rule is the aforementioned 50% vote share, distinguishing between electoral winners and losers. The running variable is the margin of victory ($M_{i}$), which is defined as the difference between the winner’s vote share and the 50% cutoff. The following models are estimated for the two different definitions of the dependent variable:

2 $$\begin{aligned} \frac{B_{i}}{A_{i}}= & {} \beta f(M_{i})+\tau R_{i}+\delta R_{i}M_{i}+{\mu \mathbf{C}_{\mathbf{i}}}+\eta _{i} \end{aligned}$$

3 $$\begin{aligned} \log B_{i}= & {} \beta f(M_{i})+\tau R_{i}+\delta R_{i}M_{i}+{\mu \mathbf{C}_{\mathbf{i}}}+\eta _{i} \end{aligned}$$

$$\begin{aligned} R_{i}=1[M_{i}\ge 0] \end{aligned}$$

for firm *i* for all bailouts received during 2008 and 2009, where $M_{i}=V_{i}-c$ is the difference between a candidate’s vote share and the 50% cutoff (or the distance between the first and second candidate when more than two candidates competed in a race), $f(\cdot )$ is a smooth continuous function of the margin of victory, $R_{i}$ is the indicator variable of whether or not a candidate won the election (representing a deterministic function of victory), and $R_{i}M_{i}$ is an interaction term. The vector of controls $\gamma C_{it}$ contains all variables mentioned in the data section measuring an individual financial institution’s market performance (leverage, deposit-to-asset ratio, earnings ratio, return on assets [ROA], Tobin’s Q), exposure to risk (the CAMELS rating), size (total assets, deposits, number of employees, employee salary), and foreclosure and subprime loan rates in their states. The functional form of $f(\cdot )$ is both linear and quadratic for the parametric estimator because I follow the suggestions of Gelman and Imbens (2018) not to enter higher-order polynomials.^11^ Given that the as-if-random assumption relies on observing the effect in narrow races, I adopt several bandwidths around the zero margin of victory threshold. The default is the $\pm 5$-percentage point (p.p.) margin above and below the threshold; however, I also enter the $\pm 1$-p.p. margin, the $\pm 3$-p.p. margin, and the $\pm 10$ p.p. margin. For an additional robustness check regarding the functional form, I rely on the Calonico et al. (2014) bias-corrected nonparametric estimator, which calculates its own optimal bandwidth (henceforth CCT).

An often-made criticism of RDD relying on close elections is that bare winners and bare losers are not necessarily interchangeable statistically. For example, Caughey and Sekhon (2011) find that bare winners raise more campaign money, while (Snyder et al. 2015) find an electoral advantage even in very close elections for incumbents and candidates whose party controls the state legislature or the governorship. The implication is that the results could be biased systematically if the sample of bare winners contained more incumbents or candidates coming from parties that controlled the legislature or held the governor’s seat. An additional concern raised by the literature is that we would observe nonrandom sorting around the threshold, questioning the key RD assumption that non-treatment covariates are continuous around the threshold.

In order to check for such bias, I present several validity tests in the Online Appendix discussing the balance of the sample with respect to incumbency status, state-level party control, and campaign funding. I find no systematic bias in favor of candidates coming from the party that controls the state-level legislature or the governorship, or in favor of candidates who spend more on their campaigns. I do find a small bias in favor of incumbents at the ±5% and ±3% electoral margins, but that bias vanishes at the most narrow ±1% margin, which represents the clearest indicator of as-if randomization. Therefore, the most unbiased results presented in Tables 2 and 3 are the ones reported for the 1% margin of electoral victory. I also perform the McCrary (2008) density test to show that no sorting is evident around the threshold that might have biased the results (reported in the Online Appendix). Finally, one additional robustness check was to vary connected firms by sizes of donations or lobbying effort, under the assumption that only firms with large contributions can have a sizeable effect on bailout distribution.

#### RDD results

**Fig. 3:**
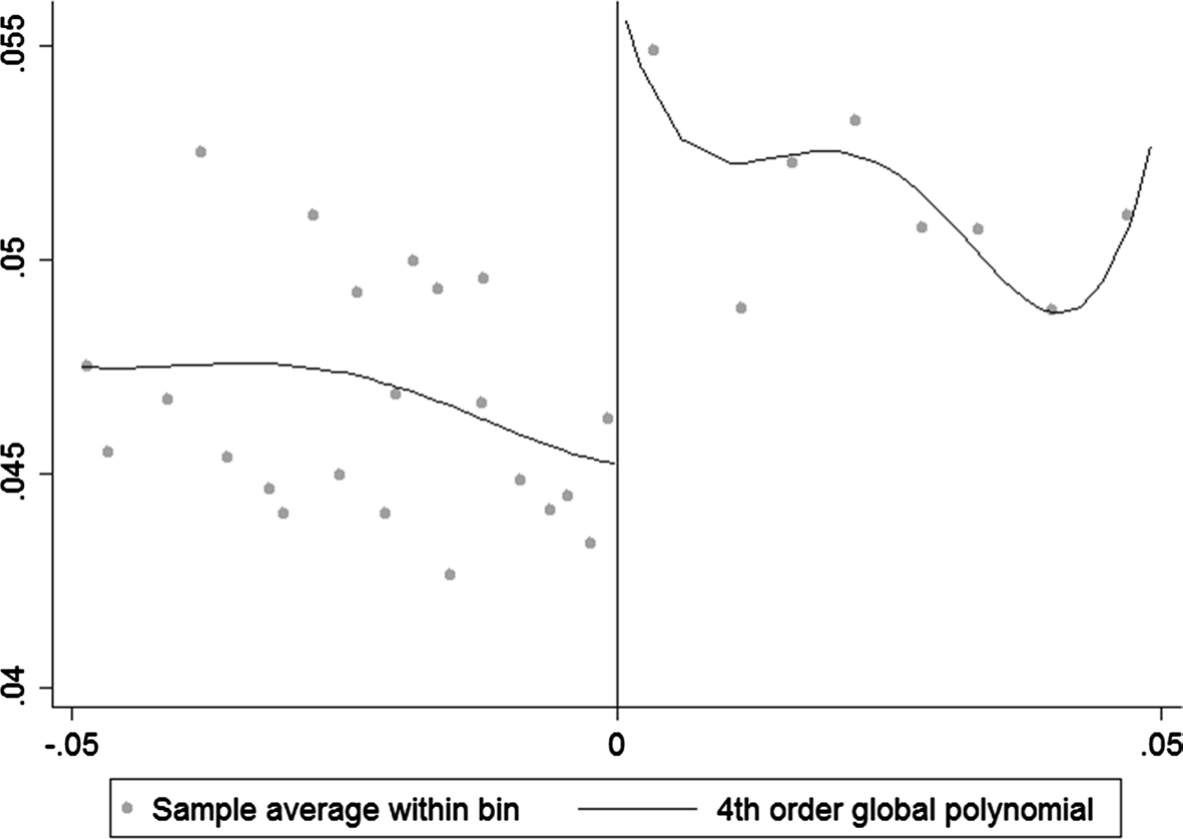
The effect of a connected politician’s victory on the share of bailouts in total assets (*y*-axis) for close races decided within a ±5-percentage-point margin of victory. The fitted lines represent a fourth-order polynomial. The polynomial is used in this case because it provides a better fit

**Fig. 4:**
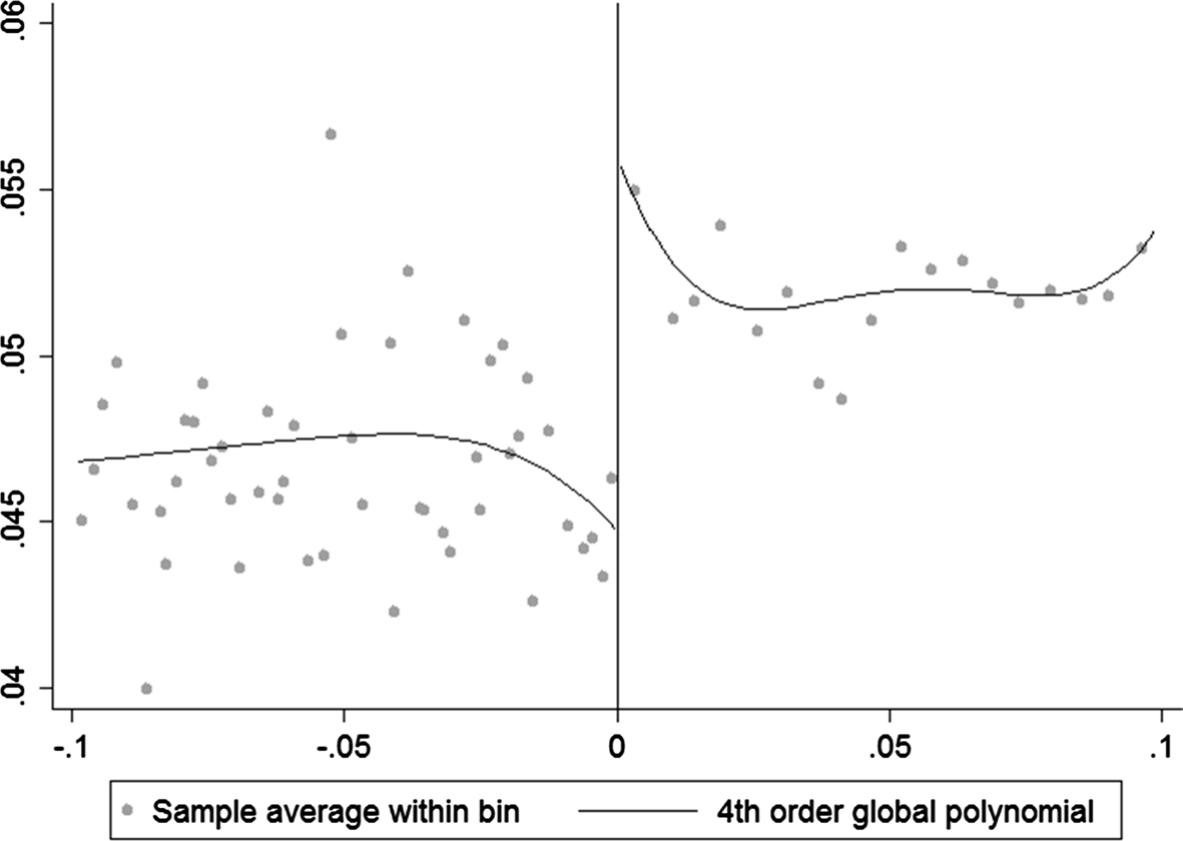
The effect of a connected political victory on the share of bailouts to total assets (*y*-axis) for close races decided within a ±10-percentage-point margin of victory. The fitted lines represent a fourth-order polynomial. The polynomial is used in this case because it provides a better fit

Before presenting the estimation results, I plot margins of victory against the share of bailouts in total assets for a 5- and a 10-percentage-point margin of victory in Figs. 3 and 4. Both figures show a clear discontinuity at the zero threshold, making it possible to estimate the local average treatment effect of a connected politician’s victory on the likelihood of receiving a larger bailout. The given discontinuity suggests that in narrow races, where politicians are statistically similar to one another, the victory of a connected politician has a positive effect on receiving a larger TARP bailout for a connected firm.

**Table 2:** Regression discontinuity design results

	Linear	Linear	Quadratic	Nonparametric
Full sample				
Effect of political connections on share of bailouts in total assets	0.0078***	0.0065***	0.0072***	0.0065***
	(0.002)	(0.0012)	(0.0017)	(0.001)
Size of effect	15.6%	13%	14.4%	13%
Bandwidth	±1	±5	±1	±8.7
Controls	Yes	Yes	Yes	Yes
*N*	56	488	56	960
Effect of political connections on log bailouts	0.016	0.035	0.065***	0.1136**
	(0.029)	(0.021)	(0.022)	(0.045)
Size of effect	n.s.	n.s.	6.7%	12%
Bandwidth	±1	±5	±1	±10
Controls	Yes	Yes	Yes	Yes
*N*	56	488	56	1090
Reduced sample				
Effect of political connections on share of bailouts in total assets	0.0041	0.0049***	0.0052**	0.0035***
	(0.0025)	(0.001)	(0.002)	(0.001)
Size of effect	n.s.	9.8%	10.4%	7%
Bandwidth	±1	±5	±1	±9
Controls	Yes	Yes	Yes	Yes
*N*	34	314	34	670
Effect of political connections on log bailouts	0.044**	0.041***	0.071***	0.101
	(0.017)	(0.013)	(0.017)	(0.057)
Size of effect	4.5%	4.2%	7.4%	n.s.
Bandwidth	±1	±5	±1	±8
Controls	Yes	Yes	Yes	Yes
*N*	34	314	34	685

RD estimates for the marginal effect of victories of connected politicians in narrow races on the allocation of bailouts, either as shares of total assets or in log terms. The upper panel presents the results for the full sample, while the lower panel presents the results for the reduced sample excluding the recipients of TARP funds before the 2008 election. For the nonparametric estimation, the optimal bandwidths are calculated using the CCT (2014) approach for bandwidth selection. Standard errors are shown in parentheses and are clustered by firm; n.s. stands for nonsignificant, so the effect size is not calculated

***Denotes significance at 1%, ** at 5%

Table 2 reports the results for Eqs. (2) and (3) for both the full and the reduced sample. Both panels show a positive causal effect of an additional connected candidate’s victory on bailout allocations to connected financial firms, holding all other recipients’ electoral performance constant. The results are confirmed partially across two samples (full and reduced), two functional forms (linear and quadratic), two choices of bandwidth size (5% and 1%), and two choices of estimation methods (parametric and nonparametric). The exceptions are results for the linear functional form in the full sample for the *Log bailouts* choice of dependent variable, the nonparametric estimates for *Log bailouts* in the reduced sample, and the 1% bandwidth size estimate for the *Bailouts to assets* choice of dependent variable in the reduced sample.

For the most narrow ±1-percentage-point bandwidth, across both functional forms, the total effect of an additional connected candidate winning a narrow race is a 13% to 15% increase in the ratio of bailouts to total assets for the connected firm in the full sample, and about 10% for the reduced sample. The effect on log bailouts is between 4.2% and 7.4% larger bailouts for connected firms in the reduced sample, and a 6.7% increase in bailouts in the full sample.

Even though the identification strategy for races decided within a 1% vote margin satisfies most adequately the as-if randomization assumption, the number of observations in that subsample suggests that we are observing only a few races across several firms (given that the unit of analysis is firm-politician). An inherent trade-off must be made here. Races decided within a 5% margin have a slight bias towards the incumbent candidate (described in the Online Appendix); however, they produce a larger sample with more races and more firms. Despite not being an ideal strategy, the 5% margin still provides a somewhat plausible identification for the total effect of connections on bailouts. In terms of which effect more accurately describes the relationship of interest, the trade-off is between a very robust identification strategy with a smaller sample and a less robust identification strategy with a larger sample, which seems to be a general problem in RDD inference.

When applying the nonparametric CCT approach for optimal bandwidth selection, the effects are similar: an additional connected politician’s victory produces a 13% larger ratio of bailouts to total assets, a 12% larger higher total bailouts received for the full sample, and slightly lower, 7% higher share of bailouts for the reduced sample. The margin of victory bandwidths chosen by the CCT approach are $\pm 8.7$ p.p. and $\pm 10$ p.p. for the full sample and $\pm 9$ p.p. and $\pm 8$ p.p. for the reduced sample. These are considerably higher than the initially chosen $\pm 1$ and $\pm 5$ p.p. bandwidths for the parametric approaches, so the next step is to verify the results by applying different sets of bandwidth sizes for the margin of victory. The results are presented in Table 3.

**Table 3:** RDD estimates for different bandwidth sizes

Bandwidth	Full sample			Reduced sample		
	Bailouts to assets	Log bailouts	*N*	Bailouts to assets	Log bailouts	*N*
±1	0.0078***	0.016	56	0.0041	0.044**	34
	(0.002)	(0.029)		(0.002)	(0.017)	
±3	0.0074***	0.046***	313	0.0063***	0.042**	201
	(0.001)	(0.016)		(0.001)	(0.02)	
±5	0.0065***	0.035	488	0.0049***	0.041***	314
	(0.001)	(0.021)		(0.001)	(0.013)	
±10	0.0051***	0.053***	1090	0.0044***	0.044***	675
	(0.001)	(0.007)		(0.001)	(0.008)	

Each row represents the estimates from a separate regression where only the main coefficient of interest is reported. Standard errors are shown in parentheses and are clustered by firm

***Denotes significance at 1%

The size of the effect changes with respect to different bandwidths, but it does not alter the overall conclusion.^12^ Each row represents the main average treatment effect for both dependent variables across four different chosen bandwidths and across both samples. In the case of bailouts to total assets, effect size increases in magnitude as we move closer to the zero threshold, which is expected given the necessary identification assumption of the RD approach (this is also suggested in Figs. 3 and 4). The effect also increases in magnitude as we approach the zero threshold for log bailouts in the reduced sample. It is not surprising to see a highly significant effect regardless of chosen bandwidth; however, it is reassuring that the effects remain strongest for narrow races where the as-good-as-random assumption is most likely to hold despite having fewer observations.

Another good robustness test for RDD is suggested by Imbens and Lemieux (2008), where instead of changing bandwidths, we change the value of the threshold, i.e., move it left and right of the original zero threshold to generate a set of placebo thresholds around which there should be no effect. Redefining the cutoff at 55% or 60% (or 45% and 40%) should not yield any effect on bailouts, at least not as a consequence of a narrow political race. Table 4 shows that this is indeed the case. In none of the four chosen placebo thresholds do I find any statistically significant effect of the margin of victory on the allocation of bailouts. This further strengthens the original findings.

**Table 4:** Placebo tests: RDD estimates across different thresholds, around the default zero threshold

Threshold	Bailouts to assets	Log bailouts
$-$10	$-$0.00005	$-$0.112
	(0.002)	(0.063)
$-$5	$-$0.0005	0.032
	(0.003)	(0.096)
$\mathbf {0}$	$\mathbf {0.0066}$***	$\mathbf {0.049}$***
	(0.0009)	(0.011)
+5	0.0028	0.009
	(0.017)	(0.05)
+10	$-$0.001	$-$0.057
	(0.002)	(0.07)

Estimation performed only for the full sample (results for the reduced sample yield an identical conclusion). The bandwidth in each case is the ±5 p.p. The results for the zero threshold are bolded. Each row represents the estimates from a separate regression where only the main coefficient of interest is reported. Standard errors are shown in parentheses and are clustered by firm

***Denotes significance at 1%

Finally, I look at whether results change in any way if I vary connected firms by size of donation or lobbying spending. I use several definitions: first I look at firms who spent at least $1 million during the 2008 cycle, then I look at firms which spent at least $100,000, and finally some relative measures of campaign donations only: share of firm’s donation to a candidate’s campaign budget, size of donation to total assets, and size of donation to total bailouts received, where in each case I take only firms above the median of the distribution for the entire variable and code them as robustly politically connected (connected conditional on size of their campaign donation or lobbying expenditure). All estimations confirm the results from Table 2. In each case they slightly reduce the dataset and hence change the sizes of the estimated effects, but in all cases the results are still significant and in the same direction. This is most likely because there is not much difference between the old and new samples across each of the newly defined metrics of connections. In other words, for financial firms which spent smaller amounts on donations and lobbying, their connected politicians did not win or lose in *close* elections. The results for this robustness test are reported in the Online Appendix.

### Instrumenting for connections using winners of close elections

The close election setting allows for one more identification strategy. The unit of analysis is now the firm, while the dependent variables are, as before, *Bailouts as share of assets* and *Log bailouts*. The treatment is the number of politicians connected to a single firm (instead of firm board members connected to politics). This new treatment variable captures the total number of politicians an individual firm lobbied, was connected to via senior executives, or donated to their campaigns in the 2008 cycle. The focus is on estimating the effect of political connections (total number of connected politicians per each firm) on relative bailouts received by the firm. However, endogeneity is likely to be a problem given that larger banks were connected to more politicians and also received larger bailouts. For example, each of the five biggest banks (Citigroup, Bank of America, J.P. Morgan, Morgan Stanley, and Wells Fargo) had over 200 connected politicians in the 2008 cycle.

For that reason I design an instrumental variable (IV) which measures the total number of connected politicians per firm who won in 2008, conditional on having won a close election. This variable is much more randomly distributed across firms, thus eliminating the upward bias of the biggest banks who had 10% to 13% of connected politicians winning a close election. The IV captures the extent to which a firm’s investment into political connections falls short of expectations on politicians winning their races due to randomness in outcomes of close elections. The assumption is that a firm investing abnormally into a politician expects a return on their investment in the form of a better bailout deal. However, there is still uncertainty regarding the politician’s electoral outcome, particularly in close races. A close race therefore provides an as-good-as-random source of variation for the explanatory variable, the number of connected politicians per each firm. The instrument derived from this relationship is the total number of connected politicians winning close elections, defined for each firm. I follow an approach similar to Clots-Figueras (2011, 2012), who uses the fraction of women who won close elections as an IV for the share of women in local legislatures in India, and Hyytinen et al. (2018), who use within-party variation in close elections in Finland as an IV for the share of municipal employees in a local legislature.

I design the IV in the following way. First, using the firm-politician RDD dataset I define three bandwidth sizes for close election margins: the 5%, 3%, and 1% bandwidth. I then look at which politician won an election and multiply it by the binary indicator of the narrow electoral margin, $C_{j}$, defined as:

4 $$\begin{aligned} C_{j}={\left\{ \begin{array}{ll} 1, &\quad {} {\text {if}}\,v_{j}\le \left| \Delta \right| ,\\ 0, &\quad {} {\text {if}}\,v_{j}>\left| \Delta \right| \end{array}\right. , } \end{aligned}$$

where $v_{j}$ is the electoral margin of victory for politician *j*, and $\Delta$ is defined as either ±1%, ±3%, or ±5%. Within such narrow electoral thresholds, candidates are assumed to be similar to each other in all unobservable characteristics. The close election setting emphasizes the impact of uncertainty on the outcome, meaning that either one of the candidates could have won, but a random factor such as luck played a role in tipping the seat from one to the other.

The instrument is then simply the sum of all connected politicians for firm *i* who won a close election within each of the three margins:

5 $$\begin{aligned} Z_{i}=\sum _{i}C_{j}W_{j} \end{aligned},$$

where $W_{j}=1$ if $v_{j}\ge 50$, and 0 otherwise (or if the distance between the first and second candidate is $\ge 0$ when more than two candidates competed in a race).

When defined this way, the instrument fully captures the random variation of the electoral outcome: $Z_{i}$ is *higher* for firms that had more politicians who got lucky on election night, and *smaller* for firms that had more politicians who were unlucky on election night. Luck here is defined as any outcome that might have tipped a close election in one or another direction (e.g., rain on election day that might have affected turnout). A firm could not foresee how many of its connected politicians would win or lose close elections, meaning that the IV is as good as exogenous over the expected outcomes. This, however, does not prove the exclusion assumption, meaning that further validity checks of the identification strategy need to be done to justify the inclusion of this instrument.

#### IV validity check

I perform two validity checks, following the logic in Clots-Figueras (2011, 2012). The main test was to compare firms where more politicians won than lost close elections to firms where more politicians lost than won close elections. That is carried out through a series of *t*-tests for each right-hand-side variable across all three bandwidths. Results are presented in Table 5. The indicator variable for performing the *t*-tests is defined as follows: all firms that had more politicians winning than losing close elections are in the treatment group, while all firms that had more politicians losing than winning close elections are in the control group. The expectation is that firms on both sides are similar to each other across all covariates, particularly size and risk exposure.

The results suggest that the samples are not fully balanced between treatment and control groups for the 5% and 3% margins, meaning that bigger and riskier firms have a larger number of politicians winning close elections (e.g., at the 5% margin there is a clear bias in favor of bigger firms). However, at the 1% electoral margin there is no such bias. That is reassuring, given that those margins suggest very close elections where chance was more likely to play a role in the outcome. Looking into the dataset, it is clear that a few large banks had more politicians winning than losing at a 5% or 3% margin; however, for really close races, the winners and losers across even the larger banks balanced out more evenly. The validity test merely suggests that one should be careful in interpreting the estimated effects at the 5% or 3% margins, as those results might still be biased due to firm size. For very close electoral races, specifically those decided within a 1% electoral margin, the bias is reduced and our estimated effects should be more precise.

**Table 5:** IV balance checks: comparing firms where more politicians won than lost a close election to firms where more politicians lost than won a close election

Covariate		±1%	±3%	±5%
Log assets	Difference in means	$-$0.544	$-$0.425	$-$0.656
	*t*-value	$-$1.59	$-$1.53	$-$2.86**
CAMELS risk rating	Difference in means	$-$0.0066	$-$0.03	$-$0.034
	*t*-value	$-$0.354	$-$2.04**	$-$2.70**
ROA	Difference in means	0.007	$-$0.004	$-$0.003
	*t*-value	1.12	$-$0.72	$-$0.604
Tobin’s Q	Difference in means	$-$0.011	$-$0.019	$-$0.011
	*t*-value	$-$0.296	$-$0.66	$-$0.45
Earnings assets	Difference in means	0.153	0.061	0.171
	*t*-value	1.708	0.82	2.82**
Leverage	Difference in means	0.027	0.061	0.127
	*t*-value	0.356	1.00	2.49**
Deposits to assets	Difference in means	$-$0.018	0.003	0.074
	*t*-value	$-$0.263	0.055	1.55
Log salaries	Difference in means	$-$0.766	$-$1.41	$-$1.168
	*t*-value	$-$0.753	$-$1.74	$-$1.67
No. of employees	Difference in means	$-$12154	$-$24711	$-$24705
	*t*-value	$-$0.922	$-$2.38**	$-$2.81**
% Foreclosures	Difference in means	0.004	$-$0.003	$-$0.0008
	*t*-value	0.998	$-$0.78	$-$0.26
% Subprime loans	Difference in means	$-$0.004	$-$0.007	$-$0.007
	*t*-value	$-$0.669	$-$1.36	$-$1.59

Table presents the results of *t*-tests performed for each covariate. The indicator variables for performing the *t*-tests put all firms who had more politicians win than lose a close election in the treatment group, and all firms who had more politicians lose than win a close election in the control group. Three indicators are defined, each for one narrow margin of victory

Additionally, I also examine whether the instrument is independent of any other firm-level characteristics that I can control for. I run ordinary least-squares (OLS) regressions with the instrument as the dependent variable and every firm-level characteristic as a right-hand-side variable. The second validity test is much weaker than the first one, given that it does not account for unobservable variables that might affect the instrument. It can, however, be useful to uncover potential bias in the estimated effect of 2SLS estimation. Similar to the results reported in Table 5, at a 1% margin there are no variables in the sample that carry any effect on the proportion of a firm’s connected politicians who won close elections. For the 5% and 3% margins there is a positive effect of asset size and a negative effect of earnings assets. The implication is that for firms with winners under 3% and 5% margins of victory, larger firms had more close electoral winners, which could result in a biased estimated effect for that specific sample. One should thus remain cautious when interpreting the estimated effects for those margins. Results of the second validity check are reported in the Online Appendix.

#### IV estimation and results

The validity checks performed present me with enough confidence to run the 2SLS estimation procedure using the instrument at hand. The reduced-form equation is estimating a local average treatment effect (LATE): the impact of a firm’s connected politicians that won close elections on the relative size of bailout funds it received.

6 $$\begin{aligned} \log B_{i}=\alpha _{0}+\tau Z_{i}+{\gamma \mathrm{C}_{\mathrm{i}}}+\epsilon _{i} \end{aligned}$$

The dependent variables are, as before, *Log bailouts* and the *Share of bailouts in total assets*, $\frac{B_{i}}{A_{i}}$ (not shown in Eq. 6). I run the 2SLS estimation for each of the two main dependent variables and present both sets of results in Table 6. The first stage for the sum of all connected politicians for firm *i* is:

7 $$\begin{aligned} \sum PolCon_{i}=\alpha _{0}+\beta Z_{i}+{\gamma \mathrm{C}_{\mathrm{i}}}+\varepsilon _{i} \end{aligned}.$$

**Table 6:** Firm-level instrumental variable results

Bandwidth	IV estimates			
	±1	±3	±5	OLS
Effect of connected politicians on share of bailouts in total assets	0.0009**	0.0007**	0.0006**	0.0006**
	(0.0004)	(0.0003)	(0.0003)	(0.0003)
Size of effect	2.3%	1.9%	1.6%	1.6%
R2	0.2161	0.2182	0.2182	0.2182
Effect of connected politicians on log bailouts	0.016***	0.018***	0.02***	0.0195***
	(0.005)	(0.006)	(0.006)	(0.006)
Size of effect	1.6%	1.8%	2.0%	1.9%
R2	0.3401	0.3402	0.3403	0.3403
First stage	30.27***	11.81***	8.17***	–
	(0.73)	(0.178)	(0.96)	
Controls	Yes	Yes	Yes	Yes
*N*	548	548	548	548

Reported are IV estimates for the average treatment effect of the number of politicians connected to the firm, instrumented by the number of the firm’s connected politicians who won in 2008, conditional on having a close election. The bandwidth size indicates how close the election was: with a ±5% margin, a ±3% margin, or a ±1% margin. The treatment variable is the number of connected politicians for each firm, and the instrument is the number of connected politicians for each firm that won a closely contested election. List of covariates is given in Table 5. Standard errors shown in parentheses and robust to heteroskedasticity

***Denotes significance at 1%, ** at 5%

Table 6 reports the IV estimates for the local average treatment effect of the number of politicians connected to a firm, instrumented by the firm’s connected politicians who won close elections, on relative bailouts the firm received. I report the results of all three 2SLS estimations, each corresponding to a different margin of close electoral victory. I also report the OLS estimates of the basic relationship of interest in the final column.

The effects from 2SLS estimation are of similar size as the OLS effects, and are statistically significant for both dependent variables and across all three electoral margins. The size of the effect is larger the narrower the electoral margin for politicians who won when the dependent variable is *Share of bailouts to total assets*; however, the effect is smaller the larger the electoral margin when the dependent variable is *Log bailouts*. The effect may seem small—it is around 2% on average across all estimations, considerably smaller than in the RDD analysis—but it is still substantial given that it captures a unit change of an additional politician on the size of bailouts received.

Validity checks reported in the previous section concluded that estimation under the 5% and 3% margins produces an unbalanced sample where larger and riskier firms are more likely to have more connected politicians winning close elections. The implication is that positive results for the 5% and 3% margins reported in Table 6 are most likely biased due to firm size (as they are for the OLS estimates). That, however, is not a problem when looking at the sample of winners at the 1% margin. The results of the IV estimation are, unsurprisingly, the most accurate for the most narrow electoral winners (just as was the case with RD estimation). The IV identification strategy implies that only very close races are truly random, i.e., those where luck plays a crucial role. Therefore, the most useful results that can give some indication of a causal effect in Table 6 are the ones reported in column (1) for very narrow races where electoral outcomes were indeed as good as randomly assigned. The size of the effect is 2.3% for *Share of bailouts to total assets*, and 1.6% for *Log bailouts*, meaning that a bank that had one additional connected politician who won a close race at a 1% margin got on average a 2.3% higher share of bailouts, or a 1.6% higher bailout package, holding other things equal.

## Discussion of results

The two different methods applied in the paper both suggest the same conclusion: political connections mattered among TARP recipients, as they increased relative bailout packages for connected firms. However, effect size differs given that each method estimated a different relationship of interest.

The RDD estimation used firm-politicians as a unit of analysis and only focused on a subsample of connected firms to generate the firm-politician unit. The running variable is the margin of victory of a connected politician who won a close election, meaning that the interpretation of the effect is whether a close electoral victory of a connected politician increased the relative bailout allocation for that politician’s connected firm. To be more precise, it is the effect of an additional connected candidate win, holding all other connected candidates’ electoral performance constant.

The IV estimation focused on firms as a unit of analysis. The main explanatory variable is the number of politicians connected to a firm. The instrument is the proportion of connected politicians who won a close race in the 2008 election. The estimated effect is the impact of having one additional connected politician who won a close election on the relative size of bailout funds received.

In both cases the dependent variables are the same, sample selection is focused only on TARP recipients, excluding any firm that did not receive TARP funds, but changes arise from units of analysis and the choices of main explanatory variables. The first obvious implication is that the estimated effects seem small. However, the RDD and IV estimates represent marginal effects of one additional connected politician. To measure their true impact, one should multiply the individual marginal effect of a connected politician to the total number of connected politicians for each firm. For example, in the IV sample, the standard deviation of connected politicians who won a close election is 11.8. Moving up by one standard deviation, i.e., lobbying or donating to 12 more politicians who would win a close election, increased the firm’s bailout funds by 19.2%, which is a much larger effect. It is easy to see how the effect grows stronger for firms which had more connected politicians, meaning that those at the upper extremes of the distribution will yield higher benefits from their political connections. The same is true for the RDD estimation.

Altogether, the results suggest that politics played an important role in the TARP allocation process. Individual members of Congress obviously did not have so much discretionary power during the process, meaning it would be wrong to conclude that the entire allocation of bailout funds was skewed based on individual political connections, or that there was a deliberate mechanism that favored some companies over others. However, in light of the evidence presented in this paper, the influence of political connections on the allocation of bailouts cannot be ignored. That does not suggest the allocation included any illicit activities, but simply that the system worked in favor of those who were well-connected. Being a member of a politician’s social network in times of crisis obviously benefited the company.

Financial crises do not happen very often, but stressful situations where quick decisions are required from policymakers are not that rare. Figuring out how policymakers make their decisions in such contexts, as well as the consequential outcomes of such decisions, can be good motivation for further research.

## Supplementary Information

Below is the link to the electronic supplementary material.

Supplementary material 1 (pdf 280 KB)

## Footnotes

1 The CAMELS rating system stands for capital adequacy, asset quality, management quality, earnings, liquidity, and sensitivity to risk as categories from which it creates a single index. The Treasury has not made the ratings public; however, I re-create them using the same indicators. See further information in the data section.

2 The CPP was an integral first part of the TARP through which the Treasury purchased $250 billion of preferred stock of financial institutions, instead of purchasing mortgage-backed securities as initially announced.

3 The EESA was a law passed by Congress in October 2008 through which Treasury Secretary Paulson created the $700 billion TARP.

4 When regulators, under persistent influence of lobbyists, start thinking like the industry they regulate.

5 All data are publicly available at www.opensecrets.org.

6 Also publicly available at https://wrds-web.wharton.upenn.edu/wrds/.

7 The level of expected lobbying in 2008 was estimated based on the lobbying series from 1998 to 2007, and applying the following exponential smoothing formula in the final stage: $E(L_{2008})=\mu L_{2007}+(1-\mu )L_{2007}^{P}$, where $\mu =0.2$, the predicted time-series value of lobbying in 2007 is $L_{2007}^{P}$, and $L_{2007}$ is the actual value of lobbying in 2007. The same calculation was performed for 2009.

8 The estimations of heterogenous treatment effects are available upon request from the author.

9 The histograms for both outcome variables are available in the Online Appendix.

10 Bank of America, Bank of New York Mellon, Citigroup, Goldman Sachs, J.P. Morgan, Morgan Stanley, State Street, and Wells Fargo. Citigroup received an addition $20 billion on November 23rd, while Bank of America received an additional $20 billion on January 16th.

11 The quadratic function is similar to equation (2), and it looks like this: $\frac{B_{i}}{A_{i}}=\tau R_{i}+\beta _{1}M_{i}+\beta _{2}M_{i}^{2}+\delta _{1}R_{i}M_{i}+\delta _{2}R_{i}M_{i}^{2}+\mu C_{i}+\eta _{i}$.

12 I have also used ±2, ±2.5, ±4, and ±7.5, and they all yield the same conclusion.
