# Supplementary material for: The politics of bailouts: Estimating the causal effects of political connections on corporate bailouts during the 2008–2009 US financial crisis
Source: Public Choice. 2021 Feb 6;189(1-2):213–38. doi: 10.1007/s11127-020-00871-w (PMC7865106; doi:10.1007/s11127-020-00871-w)
Supplement: Supplementary file 1 — Supplementary material 1 (pdf 280 KB) [file 11127_2020_871_MOESM1_ESM.pdf]

Online Appendix: The politics of bailouts

Vuk Vukovic*

November 2020

Notes on data: Definition of control variables and inclusion of the biggest banks in the sample

The following performance indicators are included in the analysis: *Leverage* (total debt divided by total assets), *Deposit-to-asset ratio* (deposits divided by assets), *Liquidity* (cash holdings divided by assets), *Earnings ratio* (earnings assets divided by total assets), *ROA* (net income divided by total assets), and *Tobin's Q* (book value of assets minus book value of equity plus market value of equity divided by book value of assets). In order to quantify the relative risk of a financial institution I follow the approach used by Duchin and Sosyura (2012) who approximate the CAMELS rating system to evaluate individual risk of a bank (because the official CAMELS ratings records are not publicly available). The CAMELS rating system has six categories which form one common indicator of risk. These include: Capital adequacy, Asset quality, Management quality, Earnings, Liquidity, and Sensitivity to market risk. I calculated *Capital adequacy* as the ratio of Tier 1 capital to net risk-weighted assets, *Asset quality* as the ratio of net losses to total loans and leases, Earnings as *ROA*, defined above, *Liquidity* as cash over deposits, and *Sensitivity to market risk* as the sum of short term assets and liabilities divided by earnings assets. I could not get any data on management quality so I omit this variable, but I still manage to construct a suitable index of risk for each institution (I cross-checked my grades with Duchin and Sosyura's (2012) published grades and the numbers are almost identical). The index is calculated as a weighted average of the five indicators multiplied by 100, and designed so that the higher the value of the index, the lower the exposure to risk of a particular financial institution.

For each absolute value variable (such as total assets or total deposit) I take its natural logarithm. In the final dataset I therefore include *Log total assets*, *Log total deposits*, *Log risk-weighted assets*, *Log ALLL* (allowance for loans and lease losses), and *Log salary*. I only take

*Department of Politics and International Relations, University of Oxford, Manor Road Building, Manor Road, Oxford OX1 3UQ. Email: vuk.vukovic@pmb.ox.ac.uk. Oraclum Intelligence Systems, 23 Arnold Close, Hauxton, Cambridge CB22 5FN. Email: vuk@oraclum.co.uk

Number of employees at face value. I use the Log ALLL variable as an additional robustness indicator for credit risk, given that this indicator measures how much reserves a bank keeps for bad debts. Finally I also included the *average foreclosure rate* (% of loans to start foreclosure in the past 18 months) and the *average subprime loan rate* (subprime loans to total loans) for the state where the financial institution was headquartered. This data was taken from the US Department of Housing and Urban Development and their Neighbourhood Stabilization Program data.

Initial regression estimations include even the big banks that were "forced" by the government to participate in the first wave of the capital purchase program (CPP) on October 28th 2018[1]. The argument for their exclusion seems to suggest that they would have received the money anyway given their too-big-to-fail status (Duchin and Sosyura 2012) and the fact that they could have been requested by the regulators to participate to send a signal to others (Solomon and Enrich 2008). However there is strong reason to believe that it was the political power of these big banks in particular that triggered such a quick response from the policymakers. According to the interviews I held from September 2017 to October 2018 with various members of the SIGTARP, the TARP oversight committee, and former employees of the Treasury, they have all confirmed that the most significant influence over the TARP allocation process at the time came from the biggest and systemically most important institutions. They were involved in the process as their executives developed close relationships with policymakers and congressmen for years. In fact, Becker and Morgenson (2009) in the New York Times and Stewart (2009) in the New Yorker report a string of events, meetings, and phone calls that took place during the peaks of the panic in September and October of 2008, all of which suggested deep connections of the nine biggest banks' CEOs with the New York Fed Chairman Timothy Geithner and Treasury Secretary Henry Paulson[2]. Their efforts were focused on figuring out the best response strategy to the ongoing crisis by drafting the TARP deal, a process that heavily involved the biggest banks' top officials. The scope of the crisis necessitated this; however it would be wrong to exclude them given their crucial impact on the very bailout deal. The fact that they were "forced" to participate was by (their own) design. However in the subsequent RDD estimation I do exclude these big banks but for different reasons. The results and the corresponding intuition are unaffected.

RDD and IV validity tests

As stated in the RDD section there are several concerns with respect to the as-good-as random allocation of politicians into bare winners and bare losers of close elections, even with very

[1] This included the following eight big banks: Bank of America, JP Morgan, Citigroup, Goldman Sachs, Morgan Stanley, Wells Fargo, Bank of New York Mellon, and State Street.

[2] The two of them in particular were instrumental in landing a $12.9bn deal for Goldman Sachs during the massive bailout of AIG (Bloomberg 2009) and were heavily criticised as being Wall Street insiders looking to strike a better deal for the banks. As regulators they were considered to be too close to those they regulated (Becker and Morgenson 2009).

small margins. Concerns arise due to a potential electoral advantage of incumbents, candidates who received more campaign donations, and candidates coming from parties controlling state legislature or the governorship.

In cases of close elections decided within the 5% margin, if a party held either the governor office or the state legislature, only in 47.5% did this help the congressmen or senator win a close election. In 52.5% of cases the candidate won a close election despite not having his or her party control the state office or legislature. There is therefore no bias arising from the candidate's party controlling the legislature or governorship. The same thing can be said of the distribution of campaign funding to close election winners and losers. Even though electoral winners got more money on average (3.2m compared to 2.7m) I ran a t-test and the difference in campaign funding between bare winners and losers was not statistically significant under any close election margin. I ran a separate t-test just for incumbents. Incumbents winning close elections within the 5% margin got less money than non-incumbents on average (2.5m compared to 3m), and the t-test did not point to any systematic difference between candidates caused by campaign funding.

Incumbents however did have a slight electoral advantage. When looking at the 5% threshold, out of all candidates who won a close election 55% were incumbents. In particular 33 incumbents out of 59 winners won a close election, while only 18 incumbents out of 62 losers lost a close election. In other words if an incumbent was running for races decided in a 5 p.p. margin in 33 out of 51 cases they won (64.7%), and in 18 out of 51 they lost (35%). This implies that there is indeed a systematic bias in favor of incumbents even in close electoral races. However things change as we get closer to the 50% cut-off. For races decided within a 3 p.p. margin the same thing happens, 65% of all candidates won, and 35% lost, however for races decided at a 1 p.p. margin, exactly half, 50% of all incumbents won, and 50% of all incumbents lost. Therefore the best as-if randomization occurs at the most narrow bandwidth that we can observe. For the 1% margin we can truly say that there is no systematic bias between bare winners and bare losers, not driven by incumbency effects, state-level party control, or campaign spending.

I then check for any potential violation of the continuity assumption that the margins of victory are continuous around the threshold, i.e. that there is no sorting around the threshold that could have biased the results. In order to check for sorting I apply the McCrary (2008) density test. The results are shown in Figure 1 for averages around the threshold. The test shows no evidence of sorting (the theta statistic is -0.086 with the standard error of 0.115 and is thus not significant).

Finally, Table 1 shows results for the donation size robustness check. It reports estimated effects for firms that spent at least $1m during the 2008 cycle. It uses the same functional forms, bandwidth sizes, non-parametric estimators, different definitions of the outcome variable, and two different samples, as was the case with the original RDD estimates reported in the paper. Compared to the original estimates the sample sizes have decreased by a few observations in each case (e.g. from 56 to 51 for the 1% bandwidth, from 488 to 456 for the 5% bandwidth in the

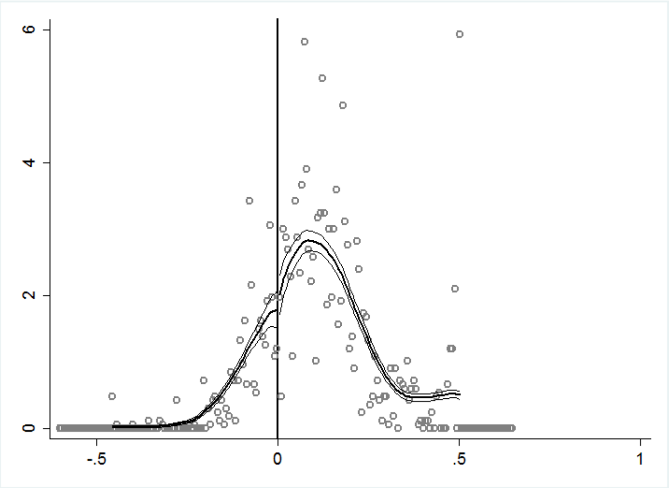

Figure 1: The McCrary (2008) density test shows no sign of sorting of the margin of victory variable around the zero threshold.

full sample), the estimated effects are slightly smaller for bailouts to assets (on average about 3 p.p. smaller) and slightly higher for the log bailouts (on average about 2 p.p. higher), while the significance levels actually increase for the log bailouts variable in the linear models of the full sample - the effects are now statistically significant at a 1% and 5% level. All other metrics of donation size reported in the paper deliver either identical results (because the sample of firms with politicians competing in close elections is the same) or slightly higher estimates with no changes in statistical significance. Full results are available upon request.

The IV validity check makes sure that the instrument is independent of any other firm-level characteristics that I can control for. Its results are presented in Table 2. It shows that there are no observed variables that carry any effect on the instrument at the 1% margin of electoral victory, but for the 5% and 3% margin there is a positive effect of asset size and a negative effect of earnings assets on the instrument. This merely implies that the results of the 2SLS estimation could result in biased estimates under those margins, however it could also be that for this specific subsample the p-values for the two variables are significant purely by chance.

Table 1: Regression discontinuity design results

	Linear	Linear	Quadratic	Non-parametric
	Full sample			
Effect of political connections on share of bailouts in total assets	0.0056** (0.002)	0.0059*** (0.0013)	0.007*** (0.0019)	0.0065*** (0.001)
Size of effect	11.2%	11.8%	14%	13%
Bandwidth	± 1	± 5	± 1	± 8.7
Controls	Yes	Yes	Yes	Yes
N	51	456	51	960
Effect of political connections on log bailouts	0.048*** (0.011)	0.048** (0.021)	0.067*** (0.014)	0.1136** (0.045)
Size of effect	4.9%	4.9%	6.9%	12%
Bandwidth	± 1	± 5	± 1	± 10
Controls	Yes	Yes	Yes	Yes
N	51	456	51	1090
	Reduced sample			
Effect of political connections on share of bailouts in total assets	0.0026 (0.0031)	0.0042*** (0.001)	0.0053** (0.0015)	0.0035*** (0.001)
Size of effect	n.s.	8.4%	10.6%	7%
Bandwidth	± 1	± 5	± 1	± 9
Controls	Yes	Yes	Yes	Yes
N	29	282	29	670
Effect of political connections on log bailouts	0.063*** (0.003)	0.065*** (0.007)	0.070*** (0.008)	0.101 (0.057)
Size of effect	6.5%	6.7%	7.25%	n.s.
Bandwidth	± 1	± 5	± 1	± 8
Controls	Yes	Yes	Yes	Yes
N	29	282	29	685

RD estimates for the marginal effect of victories of connected politicians in narrow races on the allocation of bailouts, either as shares in total assets or in log terms, when politically connected firms are defined by the size of their lobbying spending or campaign donation. Upper panel presents the results for the full sample, while the lower panel presents the results for the reduced sample where the recipients of funds before the election were excluded. For the non-parametric estimation the optimal bandwidths are calculated using the CCT (2014) approach for bandwidth selection. Standard errors shown in parentheses and are clustered by firm. n.s. stands for non-significant, so the effect size is not calculated. *** denotes significance at 1%, ** at 5%.

Table 2: IV validity check: OLS regressions of instrument against the covariates

	Dependent variable: IV		
Covariate	± 1% margin	± 3% margin	± 5% margin
Log Assets	0.213	0.908***	1.246***
	(0.212)	(0.003)	(0.002)
CAMELS risk rating	0.569	1.435	1.336
	(0.784)	(0.784)	(0.869)
ROA	-2.181	-6.015	-7.212
	(0.206)	(0.150)	(0.253)
Tobin's Q	-0.091	0.044	-0.161
	(0.885)	(0.981)	(0.957)
Earnings assets	-0.826	-3.241**	-5.335**
	(0.211)	(0.045)	(0.021)
Leverage	-0.237	-2.355	-4.015
	(0.809)	(0.451)	(0.383)
Deposits to assets	0.813	2.553	3.475
	(0.100)	(0.167)	(0.201)
Log salaries	0.002	0.049	0.178
	(0.971)	(0.701)	(0.304)
Observations	580	580	580
R squared	0.2099	0.3355	0.3661

Notes: Table presents OLS regressions of the insturmental variable defined under 3 different close election margins against each of the covariates. p-values are reported in parenthesis. Standard errors are robust for heteroskedasticity. ** denotes significance at 5%.

Figures and tables

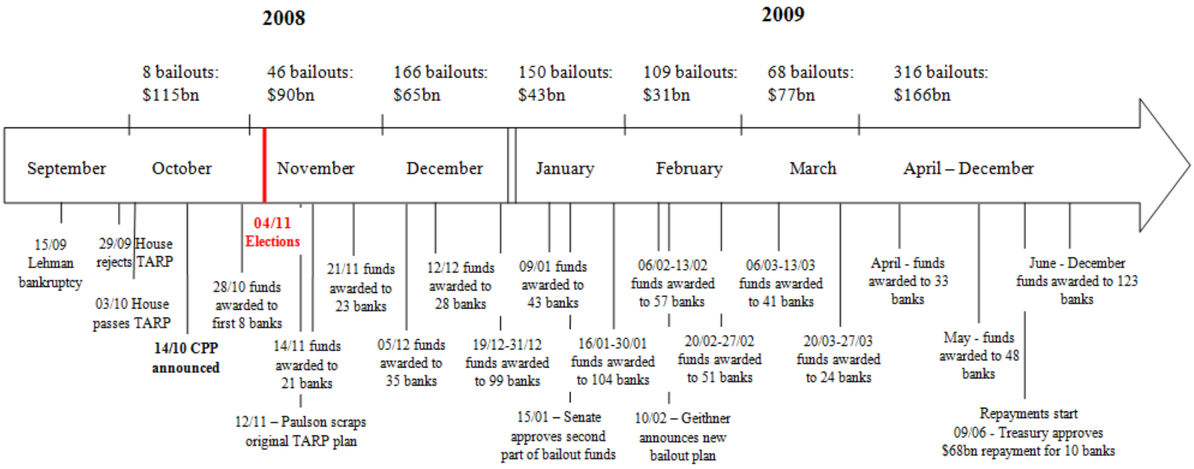

Figure 2: Timeline of the bailout allocation process. Source: Troubled Asset Relief Program Transaction Reports, March 2011, US Treasury (2013)

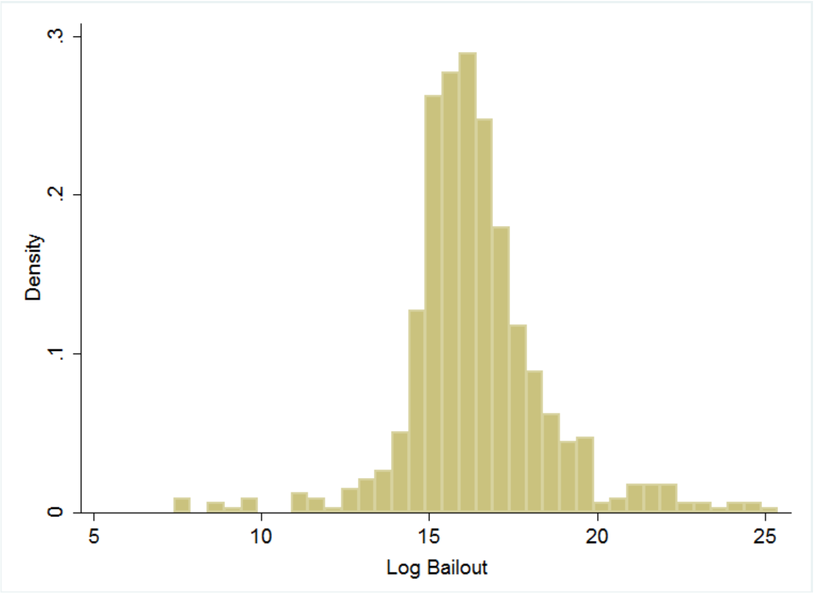

Figure 3: Histogram for the Log Bailouts outcome variable.

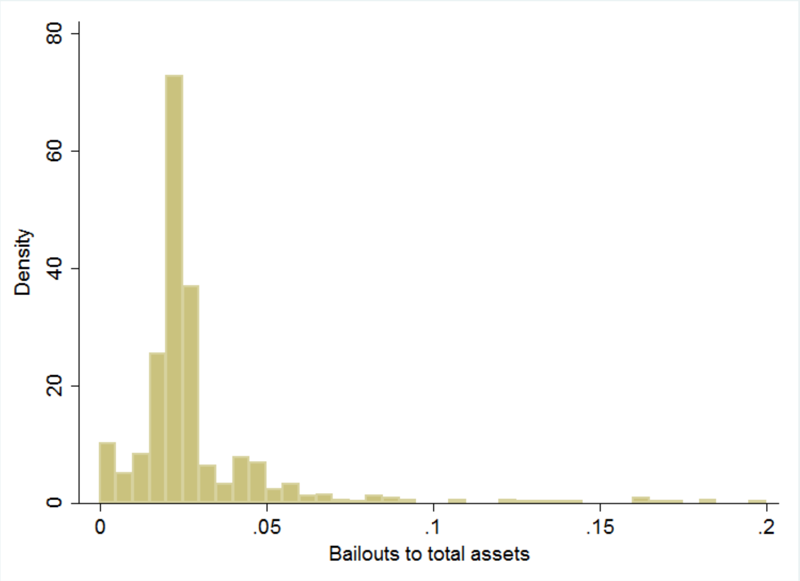

Figure 4: Histogram for the Bailouts to total assets outcome variable, excluding the outliers.

Table A1: Summary statistics for the RDD sample

Variable	Obs	Mean	Std.Dev.	Min	Max
Log bailouts	2484	22.918	0.328	0	24.94
Bailouts to total assets	2484	0.051	0.010	0	0.902
Vote share	2650	0.620	0.162	0.045	1
Won dummy	2650	0.789	0.408	0	1
Margin of victory	2650	0.120	0.162	-0.455	0.5
Cut-off	2650	0.154	0.129	0.0005	0.5
Interaction	2650	0.137	0.138	0	0.5
Foreclosure rate	2650	0.004	0.007	0.00001	0.045
Log Assets	2484	8.339	0.821	5.147	9.234
Log Deposits	2484	7.878	1.069	0	8.877
Camels rating	2484	0.128	0.057	-0.012	0.297
Capital adequacy	2484	0.093	0.032	0	0.298
ROA	2484	0.0002	0.014	-0.144	0.023
Tobin's Q	2484	0.904	0.089	0	0.998
Earnings to assets	2484	0.537	0.289	0	0.958
Allowance	2484	11.43	5.238	0	16.72
Leverage	2484	0.549	0.222	0	0.946
Deposits to assets	2484	0.508	0.213	0	0.944
Log Salaries	2484	12.82	4.075	0	16.65
Employees	2484	77396	74177	0	176003
Foreclosures in state	2484	0.041	0.011	0.021	0.070
Subprime loans in state	2484	0.12	0.017	0.059	0.141

Table A2: Summary statistics for the IV sample

Variable	Obs	Mean	Std.Dev.	Min	Max
Log bailouts	590	16.158	2.75	0	24.94
Bailouts to total assets	590	0.039	0.081	0	0.961
Treatment	590	4.63	25.15	0	263
Instrument (1%)	590	0.056	0.453	0	6
Instrument (3%)	590	0.296	1.815	0	20
Instrument (5%)	590	0.496	2.855	0	31
Won	85	24.61	46.98	0	202
Close election	85	6.035	11.8	0	57
Log Assets	583	5.73	0.808	3.994	9.234
Camels rating	583	0.0675	0.034	-0.049	0.297
ROA	583	0.0002	0.018	-0.144	0.243
Tobin's Q	582	0.899	0.058	0	0.998
Earnings to assets	582	0.87	0.13	0	0.994
Leverage	582	0.845	0.120	0	0.96
Deposits to assets	582	0.813	1.14	0	28.19
Log Salaries	583	8.91	1.86	0	16.65
Employees	583	2442	15345	0	176003
Foreclosures in state	583	0.045	0.014	0.015	0.085
Subprime loans in state	583	0.114	0.019	0.044	0.176

References

[1] Becker, J. & Morgenson, G. (2009). Geithner, Member and Overseer of Finance Club. *The New York Times*, April 26th, 2009.

[2] Duchin, R., & Sosyura, D. (2012). The politics of government investment. *Journal of Financial Economics*, 106 (2012), 24-48.

[3] McCrary, J. (2008). Manipulation of the Running Variable in the Regression Discontinuity Design: A Density Test. *Journal of Econometrics*, 142(2), 698-714.

[4] Stewart, J.B. (2009). Eight Days. The battle to save the American financial system. *The New Yorker*, September 21st, 2009.
